# Recurrent Acute Liver Failure as the First Manifestation of *ARCN1*-Associated Disorder Caused by a Terminal Exon Frameshift Variant

**DOI:** 10.3390/diagnostics16213494

**Published:** 2026-10-25

**Authors:** Varvara Shleptsova, Ekaterina Nuzhnaya, Natalia Taran, Evgeny Tatarsky, Alexandra Nikolaeva, Philipp Sviridov, Tatiana Strokova, Natalia Semenova

**Affiliations:** 1 Research Centre for Medical Genetics, 115478 Moscow, Russia; nuzhnaya@med-gen.ru (E.N.); ghetat@gmail.com (E.T.); nikolaevaepi@gmail.com (A.N.); p.sviridov@med-gen.ru (P.S.); semenova@med-gen.ru (N.S.); 2 Federal Research Centre of Nutrition, Biotechnology and Food Safety, 117296 Moscow, Russia; pknt@mail.ru (N.T.); strokova_t.v@mail.ru (T.S.)

**Keywords:** *ARCN1*, short stature-micrognathia syndrome, nonsense-mediated decay, recurrent acute liver failure, COPI coatomer, δ-COP

## Abstract

**Background**: *ARCN1*-related syndrome (short stature-micrognathia syndrome, SSMG; OMIM #617164) is an autosomal dominant disorder caused by loss-of-function variants in the *ARCN1* gene, which encodes the δ-COP (COPD) subunit of the COPI coatomer complex. The condition is classically characterized by intrauterine growth restriction (IUGR), short stature with rhizomelic shortening, microcephaly, micrognathia, facial dysmorphism, and global developmental delay. **Methods**: Clinical examination, blood tests and genetic analysis were performed. Whole-exome sequencing and Sanger analysis were conducted, followed by bioinformatics analysis and ACMG-based variant interpretation to identify and characterize the pathogenic variant. **Results**: We report a male proband with a previously undescribed variant in the *ARCN1* gene, presenting with an atypical phenotype. Notably, the patient lacked the classical syndromic features, including short stature, microcephaly, IUGR, and developmental delay. The predominant clinical manifestation was transient hepatic dysfunction occurring during febrile episodes, which resolved between acute events. On physical examination, rhizomelic shortening and dysmorphic facial features, including mild micrognathia, were found. Overall, identified symptoms were mild in severity, which may be associated with the variant’s location within the gene. **Conclusions**: We describe a novel likely pathogenic variant in the *ARCN1* gene associated with a mild phenotype of SSMG. This case expands the phenotypic spectrum of *ARCN1*-related syndrome and highlights the importance of considering this diagnosis in patients with unexplained recurrent liver injury, even in the absence of classical syndromic features.

## 1. Introduction

Phenotypic heterogeneity is a fundamental characteristic of many genetic disorders. Pleiotropy and variable expressivity can affect the clinical variability, with atypical presentation often leading to a delay in diagnosis. The median diagnostic delay across monogenic diseases is approximately 5 years, with some conditions taking much longer [1]. Approximately 38% of infants with genetic disorders in one large study had an atypical or unrecognized presentation [2]. This could prolong the diagnostic odyssey.

*ARCN1*-related syndrome (short stature-micrognathia syndrome (SSMG); OMIM: 617164) has varied phenotypic features ranging from the mild to the very severe degree. The most frequent symptoms are intrauterine growth restriction (IUGR), preterm birth, developmental delay, micrognathia and short stature, including rhizomelic shortening. Facial dysmorphism is present with a broad, high forehead, downslanting palpebral fissures, a bulbous nasal tip, hypotelorism, high-arched palate, and long philtrum [3,4,5]. Some patients experience transient liver dysfunction, glycosylation abnormalities, hypoglycemia, and cataracts [6,7,8]. Interestingly, the majority of male patients have genitourinary anomalies (microphallus, hypospadias, bifid scrotum, and cryptorchidism), while central precocious puberty has been detected in one affected female [6,9].

A total of 25 cases including the one in the present report have been reported, with six nonviable fetuses, supporting the severity status of this disease [3,4,5,6,8,9,10,11]. Moreover, severe micrognathia required a tracheostomy tube in several cases, because of respiratory failure or gastrostomy tube dependence due to severe aspiration and swallowing dysfunction [6,10].

To date, only loss-of-function variants leading to SSMG have been detected. Haploinsufficiency is a mechanism of this syndrome [4]. However, in some cases, loss-of-function variants could produce a truncated protein resulting in retention of partial function, known as hypomorphic alleles, which are associated with a significantly milder phenotypic spectrum compared to classical loss-of-function mutations [12,13,14].

The present article is intended to present a new variant of the *ARCN1* gene associated with a milder phenotype of SSMG and atypical manifestation.

## 2. Materials and Methods

The proband was clinically evaluated at the Research Centre for Medical Genetics (RCMG, Moscow) and Federal Research Centre of Nutrition, Biotechnology and Food Safety. Clinical investigations included blood tests, liver elastography and echocardiography. Informed consent was obtained from all participants.

The patient was followed-up from the first episode of liver dysfunction at 1 month of age until the time of manuscript preparation (20 months of age). Follow-up was carried out by a local pediatrician and a gastroenterologist at the Federal Research Centre of Nutrition, Biotechnology and Food Safety. Clinical and laboratory assessments were performed every 1–3 months, as well as during each acute episode and in the convalescence period. At 10 months of age, the patient was referred by the gastroenterologist to the Research Centre for Medical Genetics for genetic evaluation because of recurrent fever-associated liver dysfunction of unknown etiology.

Whole-exome sequencing (WES) was performed using the SeqCap EZ HyperCap Workflow (Roche, Vienna, Austria). Data were processed with BaseSpace software v.7.30.0 and aligned reads were visualized in Integrative Genomics Viewer v2.19.8. Variant classification followed ACMG/AMP criteria (ACMG—American College of Medical Genetics and Genomics; AMP—Association for Molecular Pathology) and Sanger sequencing was used for validation [15].

For the description of phenotypic abnormalities encountered in human disease, a standardized vocabulary (The Human Phenotype Ontology [HPO]) was used. Each term in HPO refers to a specific manifestation of a disease and has a unique numerical identifier [16].

Three-dimensional structural models of wild-type δ-COP (COPD) encoded by the *ARCN1* gene, the predicted *ARCN1* p.(Ile445MetfsTer41) mutant product, and the COPD–COPB1 complex were generated using AlphaFold Server. The mutant protein sequence was generated according to the predicted frameshift, replacing the native C-terminal COPD sequence from Ile445 onward with the newly encoded 46-amino-acid sequence followed by a premature termination codon. Predicted structures were inspected and visualized in PyMOL v2.5.8 (Schrödinger, LLC, New York, NY, USA).

## 3. Results

### 3.1. Clinical Evaluation

The patient is the second child of nonconsanguineous parents. The boy was delivered prematurely after 36.5 weeks of pregnancy. Birth weight was 2890 g (+0.38 standard deviation score [SDS]), length was 49 cm (+0.74 SDS), and Apgar scores were 8/9. The perinatal period was unremarkable, and he was discharged home at the appropriate time. Newborn screening (36 inherited diseases) was unremarkable. Echocardiography at the age of 6 months revealed a patent foramen ovale and an accessory chord in the left ventricular cavity, both of which persisted throughout the entire period of observation.

At the age of one month the proband had a first episode of acute upper respiratory tract infection (URTI)—acute bronchitis, with fever associated with jaundice without itching. A blood test showed mild elevation in transaminases (Table 1). Infective liver diseases were excluded. The patient demonstrated a marked therapeutic benefit from the combination of antimicrobial therapy and inhaled corticosteroids. No special liver-supportive therapy was performed.

The next episode of fever and jaundice with URTI occurred at 7 months of age. A more marked increase in aspartate aminotransferase (AST), alanine aminotransferase (ALT) and alkaline phosphatase (ALP) was observed. The hyperbilirubinaemia was predominantly due to the conjugated fraction (Table 1). After investigation, deficiency in α1-antitrypsin (1833 mg/L [normal value: 900–2000 mg/L]) was excluded. Liver fibrosis was detected by ultrasound testing and liver elastography (F3 METAVIR). Hepatoprotective treatment of ursodeoxycholic acid and essential phospholipids was added to antibiotic treatment with clinical improvement.

The third episode of hepatic dysfunction, which occurred at 10 months of age in association with URTI, was characterized by more pronounced cytolysis, whereas cholestasis was less marked than during the second episode at 7 months of age (Table 1). Physical examination revealed hepatomegaly and yellowish discolouration of the palmar skin without scleral icterus. Of note, serum total bilirubin remained within the reference range at that time (9.6 μmol/L; Table 1). Taken together, the normal bilirubin level and the absence of scleral icterus indicate that the palmar discolouration was not attributable to hyperbilirubinaemia but was most likely due to carotenoderma, a benign condition commonly observed in infants receiving carotene-rich complementary foods.

Between episodes, these parameters gradually decreased, although transaminase levels did not always return to the reference range (Table 1).

The total protein remained in the normal range at all checkpoints. Interestingly, hypercholesterolemia and hypoglycemia during the episodes of the URTIs were noted with normalization in each recovery period.

It should be noted that a significant elevation in bile acids (224.2 μmol/L [normal value: 0–10 μmol/L]) was detected at second episode of URTI (patient’s age—7 months). Bile acids were measured only at 7, 10 (10.74 μmol/L) and 16 months of age (5.45 μmol/L); therefore, this marked elevation was recorded only once.

**Figure 1:**
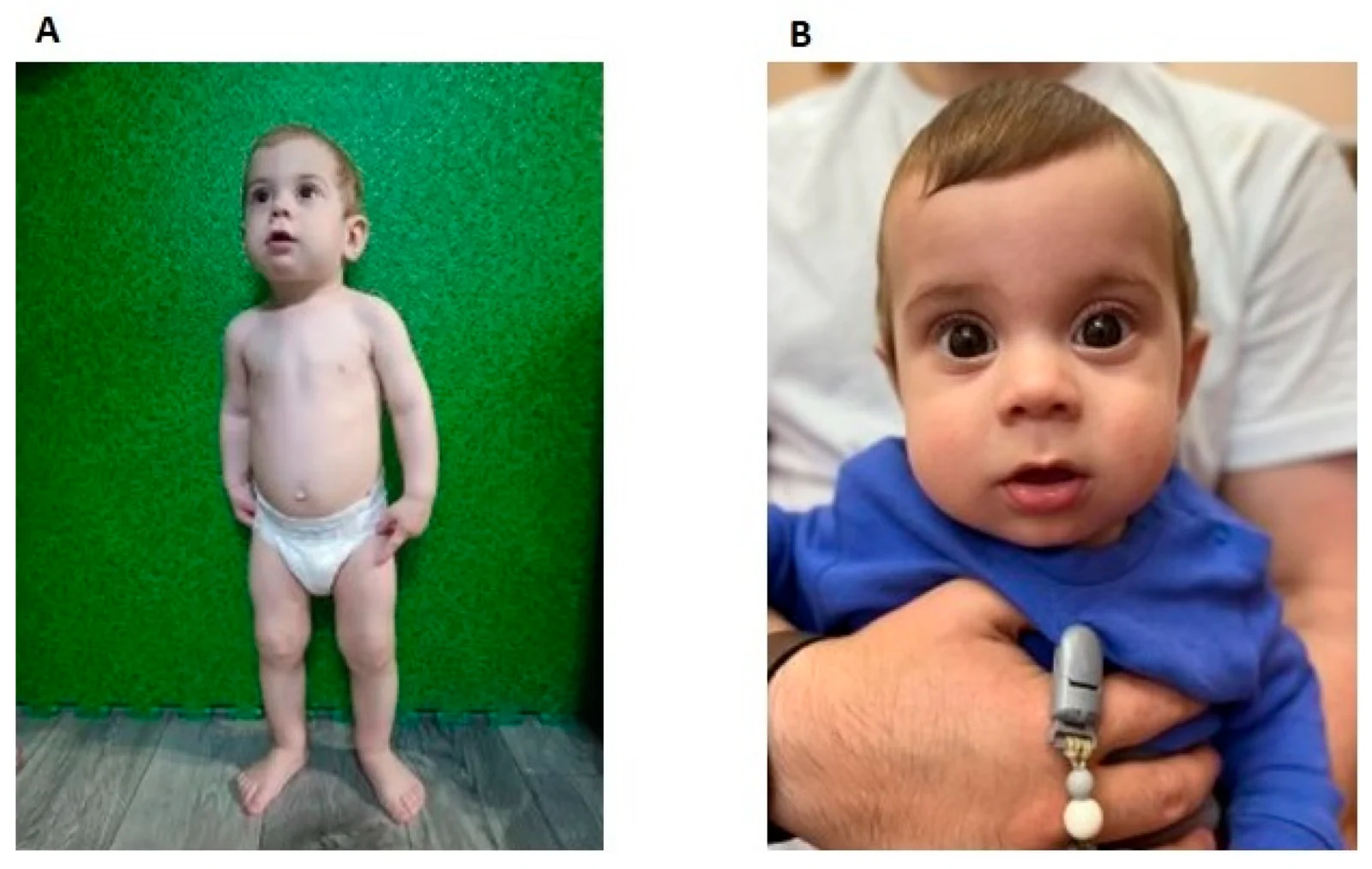
Clinical features of the patient. (**A**) Full-length view (© 2026 Nano Banana AI v.2.1 was used to hide the genitals). A mild rhizomelic shortening of the limbs and micrognathia is noted. (**B**) Full forward facial view. Facial dysmorphism including a prominent forehead, periorbital edema, hypotelorism, strabismus, bulbous nasal tip, and long philtrum is presented.

On physical examination (Figure 1), rhizomelic shortening (HP:0004991) and facial dysmorphism, including a prominent forehead (HP:0011220), periorbital edema (HP:0100539), hypotelorism (HP:0000601), strabismus (HP:0000486), bulbous nasal tip (HP:0000414), micrognathia (HP:0000347), large ears (HP:0000400), preauricular skin tag (HP:0000384), and long philtrum (HP:0000343) were found.

His developmental milestones and learning to roll, sit, crawl and walk all conformed with his age.

### 3.2. Genetic Analysis

**Figure 2:**
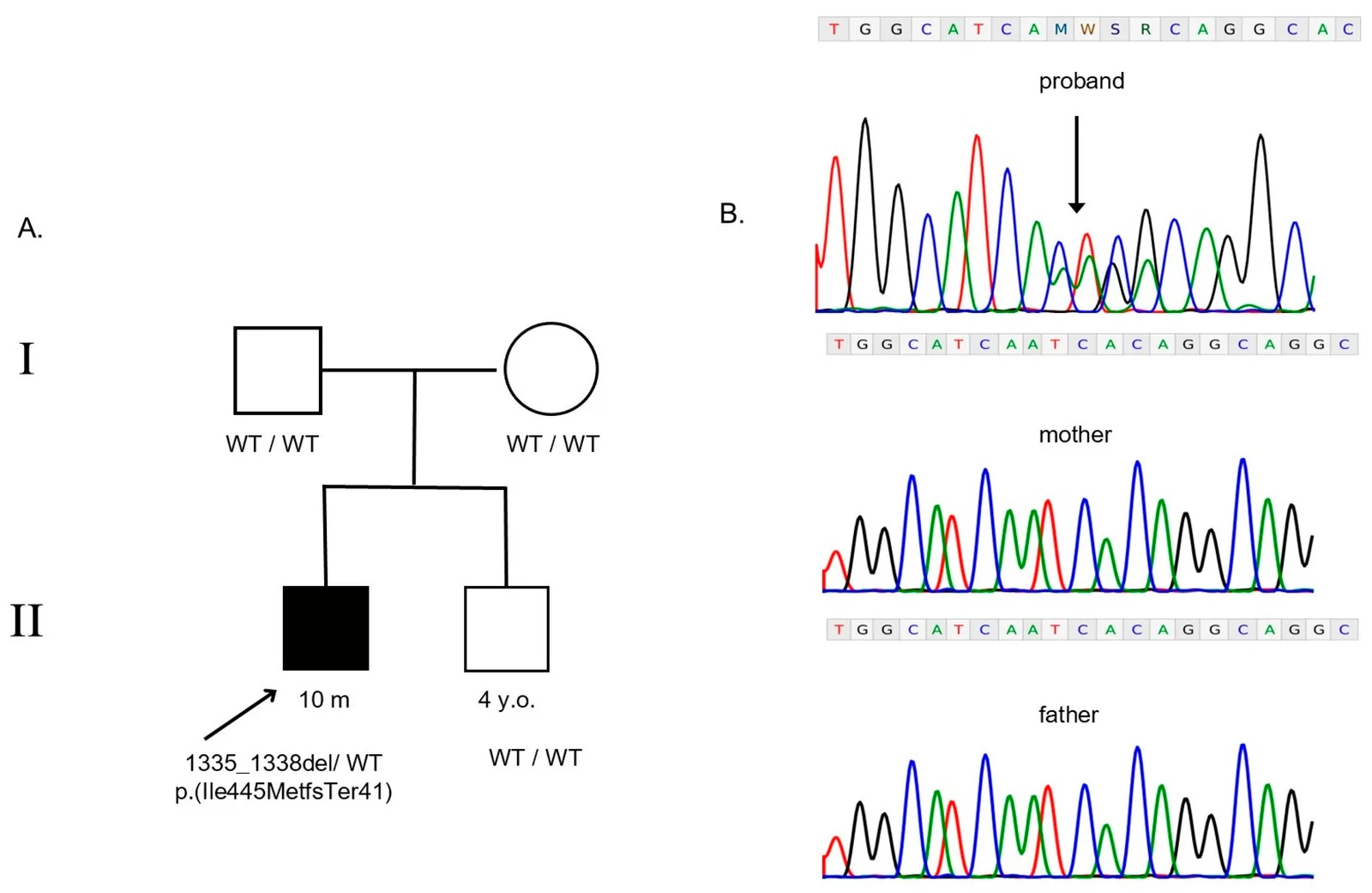
Family pedigree (**A**), WT—wild type; results of Sanger sequencing (**B**). I—first generation; II—second generation. The proband carries a likely pathogenic de novo variant in *ARCN1*. Segregation analysis confirmed that the variant was absent in both parents.

WES was performed and a previously undescribed variant in the *ARCN1* gene in the heterozygous state, NM_001655.5:c.1335_1338del, p.(Ile445MetfsTer41), was found. Sanger sequencing confirmed this change to be present only in the proband and not the parents (Figure 2). No other pathogenic or likely pathogenic variants in genes associated with hereditary hyperbilirubinaemia were identified in WES data. The *UGT1A1* promoter TA-repeat polymorphism associated with Gilbert syndrome was not assessed, as the clinical course was not suggestive of this condition.

According to ACMG/AMP guidelines [15], the variant NM_001655.5:c.1335_1338del, p.(Ile445MetfsTer41), in the *ARCN1* gene meets criteria PM2, PS2. Based on these criteria, this variant is classified as likely pathogenic. Pathogenic and likely pathogenic heterozygous variants in *ARCN1* cause SSMG (OMIM #617164). This disease is characterized by IUGR, micrognathia, rhizomelic shortening, and facial dysmorphism, including a prominent forehead, bulbous nasal tip, and other residual symptoms, including fever-related AST and ALT elevations [4,5].

This variant was evaluated as the causative variant in accordance with the clinical symptoms, laboratory and investigational results, and genotype analysis.

### 3.3. Structural Modelling of the ARCN1 p.(Ile445MetfsTer41) Variant

The *ARCN1* gene (Archain 1) encodes the delta subunit of the COPI (Coat Protein Complex I)—COPD, which is essential for retrograde transport—moving proteins from the Golgi apparatus back to the endoplasmic reticulum (ER) [17,18]. The *ARCN1* gene is located on chromosome 11q23.3 and spans approximately 30.6 kb of genomic DNA (GRCh38, chr11:118,572,409–118,603,033). According to MANE Select (NM_001655.5; ENST00000264028), *ARCN1* consists of 10 exons [19]. COPD consists of the N-terminal longin domain (LD), α-helical linker region and C-terminal μ-homology domain (MHD) [20,21] and contains 511 amino acids [22].

**Figure 3:**
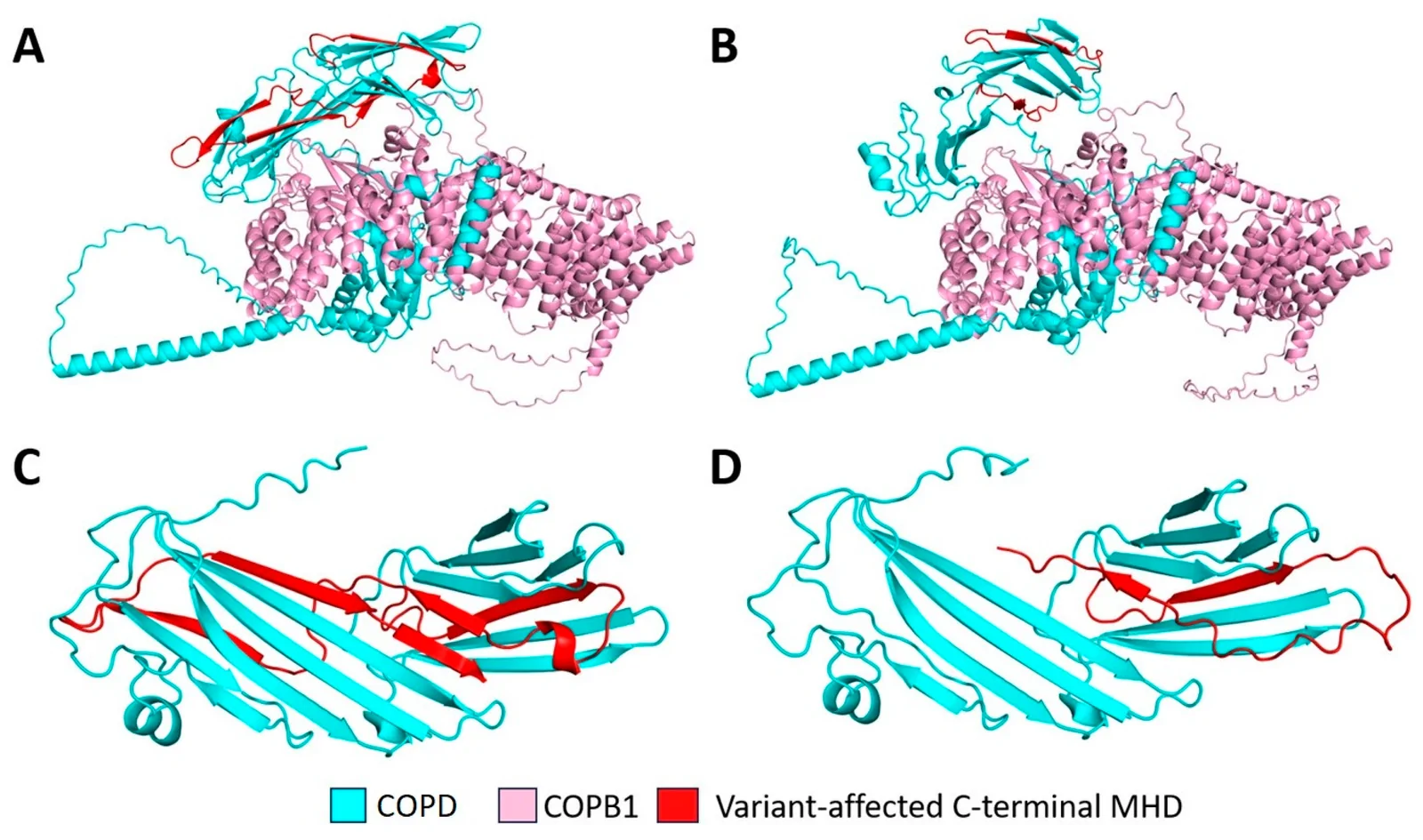
Predicted structural effect of the *ARCN1* p.(Ile445MetfsTer41) variant in the COPD–COPB1 complex. Three-dimensional structural modelling of the wild-type and predicted mutant COPD–COPB1 complexes. (**A**) Wild-type COPD–COPB1 complex. (**B**) Predicted COPD–COPB1 mutant complex. (**C**) Close-up view of the wild-type COPD μ-homology domain (MHD). (**D**) Close-up view of the predicted mutant MHD. COPD is shown in cyan, COPB1 in pale pink, and the variant-affected C-terminal region of COPD is highlighted in red.

To assess the possible structural consequences of the *ARCN1* p.(Ile445MetfsTer41) variant, three-dimensional modelling of the wild-type and predicted mutant COPD–COPB1 complexes was performed (Figure 3). The variant affects the distal part of the MHD of COPD. In the wild-type model, this region contributes to a β-sheet-rich portion of the MHD.

According to the predicted mutant structure, the newly encoded 46-amino-acid C-terminal sequence partially reproduces a β-sheet-like arrangement in one region of the affected domain. However, this predicted structural preservation is incomplete: in another region of the distal MHD, β-strand elements present in the wild-type protein are lost due to truncation and replacement of the native C-terminal sequence. The affected region is not located at the main predicted COPD–COPB1 interface, suggesting that the variant may primarily disturb the local architecture of the distal MHD rather than directly disrupt COPD–COPB1 binding.

## 4. Discussion

The reported case showed a milder phenotype of SSMG compared with those described in previous publications [3,4,5,6,7,8,9,10,11]. The data is summarized in Table 2. In spite of preterm birth, no severe postnatal complications, such as respiratory distress syndrome necessitating tracheostomy tube or feeding difficulties requiring nasogastric tube placement, were observed. These serious conditions are commonly associated with severe micrognathia which has been present in the majority of patients [3,6,11]. In the present case, however, the micrognathia was mild in severity. During physical examination, rhizomelic shortening and facial dysmorphism were found (Table 2).

The disease manifested with fever-related transient transaminase elevation and gave recurrent acute liver failure (RALF). Table 3 presents the hepatic dysfunction in more detail. This symptom was observed in approximately half of the patients with SSMG. The observed percentage may potentially be an underestimate, as not all patients underwent the longitudinal biochemical assessment, hepatic imaging and histopathological assessment.

Interestingly, in our patient, a progressive worsening of transaminase levels (ALT/AST) with each episode was observed (Table 1). The cumulative effect of recurrent hepatic injury may lead to more extensive damage, particularly in a liver with established fibrosis [23,24].

The pathogenesis of liver dysfunction with *ARCN1* gene mutation is based on impairment of vesicular transport leading to intracellular accumulation of proteins, and triggers ER stress [25]. Hepatocytes are particularly sensitive to these intracellular alterations, resulting in cytolytic syndrome (elevation in AST, ALT) and cholestasis (elevation in ALP, GGT) [26,27]. Liver structure could suffer as well, leading to subsequent ongoing hepatitis and fibrosis. As previously reported, neonatal giant cell hepatitis was detected in the three patients (who underwent liver biopsy) who had initially presented with cholestasis. Fibrosis was registered by elastography in two patients at the time of having a fever-related episode, one of these being our patient described here (Table 3).

The *ARCN1* gene (located at chromosome 11q23.3) encodes the δ-COP (COPD) subunit of the heptameric COPI coatomer, which includes also α-COP, β-COP, β′-COP, ε-COP, γ-COP and ξ-COP. COPI plays a key role in vesicle trafficking between Golgi stacks and in retrograde transport of both soluble and membrane-bound proteins from the Golgi to the ER [17,28]. Loss-of-function (LoF) variants in the *ARCN1* gene lead to misfolding of proteins and trigger ER stress. Furthermore, the *ARCN1*-associated defect in COPI-mediated type I collagen transport is thought to lead to reduced collagen secretion and to underlie the skeletal abnormalities observed in SSMG [4].

To date, RALF and skeletal abnormalities were described in some other monogenic conditions associated with *NBAS*, *SCYL1*, and *RINT1* genes [29,30]. All of them are involved in vesicular transport between the ER and Golgi apparatus. Thus, disruption in these genes provides similar symptoms. Interestingly, the frequency and severity of liver crises in *NBAS* and *RINT1*-associated diseases decrease with age, but patients with *SCYL1*-associated disease present with a progressive, cholestatic course [31]. In previous reports of *ARCN1*-related syndrome, progression of hepatic dysfunction with recurrent infectious episodes has not been described. RALF was detected only in children (9 months–7.5 years) and long-term follow-up was not performed. Notably, there were adult patients with SSMG without registered liver abnormalities [4,6]. Thus, it could be assumed that there have not been any severe or life-threatening hepatic events associated with SSMG, though the further investigations are warranted [5,6].

**Figure 4:**
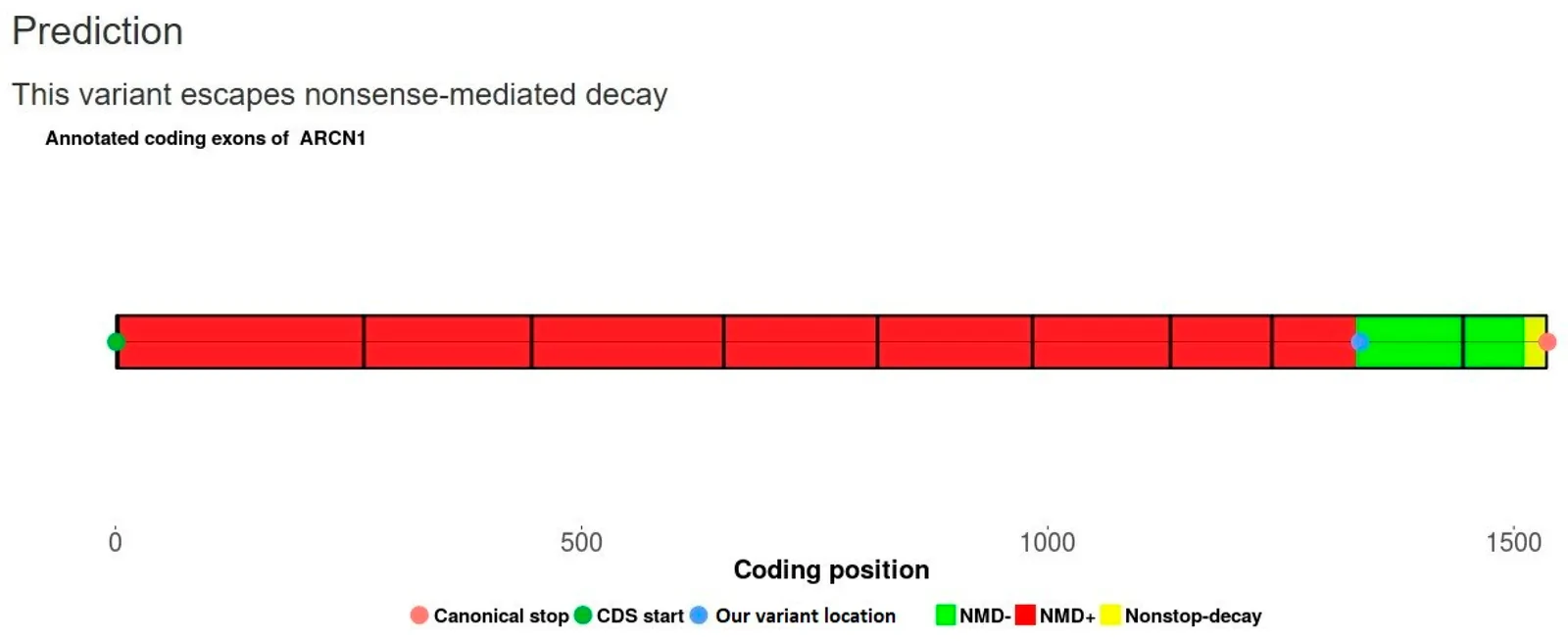
NMD prediction of NM_001655.5:c.1335_1338del in the *ARCN1* gene by NMDEscPredictor (utilizing the 50 bp rule to predict if a frameshift variant is predicted to escape nonsense-mediated decay).

Due to the mild nature of the symptoms in the patient described here, genotype–phenotype correlations should be evaluated. According to genetic analysis, the reported gene variant, which is located in exon 9, results in a frameshift with termination 41 amino acids after the deletion. This termination is located in exon 10 (the last exon). According to gnomAD v4.1.0 gene *ARCN1* is intolerant to LoF variants (pLI:1.00; LOEUF: 0.145). The DECIPHER database predicts this variant to be located within the escape of nonsense-mediated decay (NMD) region. These data are confirmed by other NMD predictor tools: NMDetective-AI [32] and NMDEscPredictor [33] (Figure 4).

Haploinsufficiency of *ARCN1* is confirmed as the pathogenic mechanism in *ARCN1*-related syndrome mediated by nonsense, frameshift, and splice-altering variants [6]. It was considered that only missense variants are associated with milder forms of SSMG [9]. However, the frameshift variant reported here, by leading to NMD escape, could produce a truncated protein [34,35,36].

For visualization of a truncated protein, three-dimensional modelling was performed. The *ARCN1* p.(Ile445MetfsTer41) variant affects the distal part of the C-terminal μ-homology domain of COPD. Although the variant is located near the C terminus, structural modelling suggests that the affected region is not a functionally neutral terminal tail, but part of a structured β-sheet-rich domain. The predicted mutant protein contains a newly encoded C-terminal sequence that may partially mimic the β-sheet arrangement of the wild-type protein in one part of the domain. However, this apparent structural preservation is incomplete, as other β-strand elements of the native C-terminal MHD are lost. Therefore, the variant may not fully abolish the COPD structure, but may selectively disturb the architecture and interaction surface of the distal MHD.

This model may help explain the relatively mild clinical presentation. Because the variant escapes nonsense-mediated decay and preserves the N-terminal region and a substantial part of the MHD, it may produce a partially functional protein rather than a complete loss-of-function allele. The partial retention of the β-sheet-like structure in the predicted mutant product is consistent with a possible hypomorphic effect. At the same time, loss of part of the native C-terminal β-sheet architecture may impair specific MHD-mediated interactions involved in COPI-dependent trafficking. Such a partial defect could be sufficient to affect sensitive developmental and secretory processes, including collagen transport/secretion, while preserving enough basal COPI function to result in a milder phenotype than more disruptive *ARCN1* variants. Indeed, Arakel et al. showed that the MHD domain is not essential for the core function of COPI, but instead plays a supportive role for COPI-mediated transport [21]. Although this observation was made in yeast, it should be noted that the *ARCN1* gene is highly conserved across eukaryotes [37,38]. The MHD domain provides specific protein-interaction plasma-membrane trafficking and increases protein transport to the plasma membrane [39]. Therefore, changes in MHD domain could lead to a partial reduction, but not a complete loss, of COPI function.

## 5. Conclusions

This article describes a novel likely pathogenic variant in the *ARCN1* gene leading to frameshift deletion. Due to strong genotype–phenotype correlation this variant was deemed as causative for SSMG even despite the minimal symptom expression. The relatively mild phenotype is thought to be associated with location in the NMD-escaping area of the gene, such that a truncated protein with partial functional ability was formed. However, further investigations of the potential effect of genetic and environmental factors on the phenotype are required.

Here, SSMG manifested with transient elevation in hepatic enzymes, but without the classical symptoms of short stature, intrauterine growth delay and global development delay. Some of the RALFs in infancy and childhood could be a severe, life-threatening condition leading to serious outcomes. *ARCN1*-related syndrome should therefore be considered in the differential diagnosis of infants with recurrent fever-associated liver dysfunction, even in the absence of short stature or developmental delay. Careful assessment for subtle skeletal and craniofacial features may prompt earlier genetic testing.

## Figures and Tables

**Table 1:** Blood test parameters throughout observation period.

Variable (Normal Values)	URTI, 1 Month	URTI, 7 Months	Convalescence, 8 Months	Healthy, 9 Months	URTI, 10 Months	Convalescence, 12 Months	Healthy, 16 Months
AST, U/L (4–40)	65.30	820	319	52.7	1279	231	40.8
ALT, U/L (4–40)	26.50	598.9	142.4	29.8	675	265.9	41
GGT, U/L (0–6 mo: <204 U/L 6 mo–1 y: <34 U/L; 1–3 y: <18 U/L)	185.50	146.2	68.9	22.4	68.4	18	13.8
ALP, U/L (<400)	370.00	2442	350.3	179	561.00	310	227.4
Total bilirubin, μmol/L (5–21)	137.80	201.1	18.77	7.82	9.6	9.7	7.5
Conjugated bilirubin, μmol/L (<5)	8.09	147.2	-	2.6	5	4.7	1.9
Cholesterol, mmol/L (3.2–5.2)	-	7.31	5.3	3.5	5.51	1.94	3.03
Glucose, mmol/L (3.9–5.8)	3.89	3.54	4.2	4.18	3.74	2.9	4.15
Total protein, g/L (51–73)	63.6	60.5	66.2	67.9	61	66	61.2
INR (0.88–1.1)	0.98	0.99	0.96	1.21	-	-	1.13

URTI—upper respiratory tract infection; AST—aspartate aminotransferase; ALT—alanine aminotransferase; GGT–glutamyltranspeptidase; mo—month(s); y—year(s); ALP—alkaline phosphatase; INR—international normalized ratio.

**Table 2:** Clinical features of our case and previous reported cases.

Symptoms	Our Case	Patients (%) ^1^	Fetal Cases (%)	Total (%)
Micrognathia	+	19/19 (100.0)	6/6 (100.0)	25/25 (100.0)
Short stature	−	17/19 (89.5)	1/6 (16.7)	18/25 (72.0)
IUGR	−	17/19 (89.5)	6/6 (100.0)	23/25 (92.0)
Preterm birth	+	15/19 (78.9)	NA	16/19 (84.2)
Facial dysmorphisms	+	12/19 (63.2)	1/6 (16.7)	14/25 (56.0)
Developmental delay	−	12/19 (63.2)	NA	12/19 (63.2)
Rhizomelic shortening	+	11/19 (57.9)	6/6 (100.0)	18/25 (72.0)
Microcephaly	−	9/19 (47.4)	5/6 (83.3)	14/25 (56.0)
Liver function abnormalities	+	9/19 (47.4)	NA	9/19 (47.4)
Visual impairment	−	6/19 (31.6)	NA	6/19 (31.6)
Congenital heart disease	+	3/19 (15.8)	-	4/19 (21.1)
Genitourinary anomalies in males	−	4/8 (50.0)	3/3 (100.0)	7/11 (63.6)

^1^ Column contains total number of patients including present case [3,4,5,6,7,8,9,10,11]. IUGR—intrauterine growth restriction; NA—not applicable.

**Table 3:** Hepatic changes in SSMG patients.

Group	Symptom	Our Case	Previous Cases	Total of Patients with SSMG * (%)
Changes in liver enzymes	Transient hepatic transaminase elevation (increase in AST, ALT)	+	8	9/19 (47.4)
	Transient cholestasis (increase in ALP, GGT)	+	3	4/19 (21.1)
Structural changes	Neonatal giant cell hepatitis	−	3	3/19 (15.8)
	Hepatoblastoma	−	1	1/19 (5.3)
	Hepatomegaly	+	1	2/19 (10.5)
	Fibrosis	+	1	2/19 (10.5)

* These patients were described by Reunert at al., Ritter at al. and Kazakova et al. [3,6,8]. AST—aspartate aminotransferase; ALT—alanine aminotransferase; ALP—alkaline phosphatase; GGT—glutamyltranspeptidase.

## Data Availability

The data supporting the findings of this study are available from the corresponding author upon reasonable request.

## Abbreviations

The following abbreviations are used in this manuscript:

ACMG	American College of Medical Genetics and Genomics
ALP	Alkaline phosphatase
ALT	Alanine aminotransferase
AMP	Association for Molecular Pathology
AST	Aspartate aminotransferase
ER	Endoplasmic reticulum
GGT	Glutamyltranspeptidase
HPO	Human Phenotype Ontology
INR	International normalized ratio
IUGR	Intrauterine growth restriction
LoF	Loss-of-Function
MHD	μ-homology domain
NMD	Nonsense-mediated decay
RALF	Recurrent acute liver failure
SDS	Standard Deviation Score
SSMG	Short stature-micrognathia syndrome
URTI	Upper respiratory tract infection
WES	Whole-exome sequencing
WGS	Whole-genome sequencing
